# The 6th Computational Structural Bioinformatics Workshop

**DOI:** 10.1186/1472-6807-13-S1-I1

**Published:** 2013-11-08

**Authors:** Jing He, Amarda Shehu, Nurit Haspel, Brian Chen

**Affiliations:** 1 Department of Computer Science, Old Dominion University, Norfolk, VA 23529, USA; 2 Department of Computer Science, George Mason University, Fairfax, VA, 22030, USA; 3 Department of Computer Science, University of Massachusetts, Boston, MA, 02125, USA; 4 Department of Computer Science and Engineering, Lehigh University, Bethlehem, PA, 18015, USA

## 

The rapid accumulation of macromolecular structures presents a unique set of challenges and opportunities in the analysis, comparison, modeling, and prediction of macromolecular structures and interactions. The 6th Computational Structural Bioinformatics Workshop (CSBW) was held in Philadelphia on October 4, 2012. This issue includes eleven papers selected from the work presented at the CSBW of 2012. In "Four-body atomic potential for modeling protein-ligand binding affinity: application to enzyme-inhibitor binding energy prediction", a predictive model of free-energy was built to evaluate the binding between a protein and a ligand. In "Unbiased, scalable sampling of protein loop conformations from probabilistic priors", a new Markov chain Monte Carlo algorithm was proposed to generate unbiased conformations of closed protein loops from probabilistic priors. In "Enhancement of accuracy and efficiency for RNA secondary structure prediction by sequence segmentation and MapReduce", authors demonstrate an enhanced method by cutting a long RNA sequence into smaller chunks at strategically selected points, and distributing the tasks to multiple processors. In "A population-based evolutionary search approach to the multiple minima problem in de novo protein structure prediction", the authors present an evolutionary search algorithm to obtain a discrete representation of the protein energy surface in terms of an ensemble of conformations representing local energy minima. In "Estimating loop length from Cryo-EM images at medium resolutions", the authors developed a computational geometry method to simplify the points along the skeleton to measure loop length in 3D images. The paper "A conservation and rigidity based method for detecting critical protein residues" presents a method that combines the rigidity and the evolutionary conservation in detection of the critical residues. In "A conservation and biophysics guided stochastic approach to refining docked multimeric proteins", the authors introduce a refinement method that accepts complexes consisting of any number of monomeric units. In "Elucidating the ensemble of functionally-relevant transitions in protein systems with a robotics-inspired method," the authors present a robotics-inspired tree-based method to sample energetically-credible conformational pathways connecting diverse functional states in multimodal proteins. The paper "An aggregate analysis of many predicted structures to reduce errors in protein structure comparison caused by conformational flexibility" applies protein structure prediction algorithms to enhance the classification of homologous proteins according to their binding preferences. In "DINC: A new AutoDock-based protocol for docking large ligands", an enhanced method was demonstrated for docking large ligands. The paper "Modeling protein conformational transitions by a combination of coarse-grained normal mode analysis and robotics-inspired methods" presents an efficient approach involving robotics concepts.

## Competing interests

The authors declare that they have no competing interests.

